# Messenger RNA translation enhancement by immune evasion proteins: a comparative study between EKB (vaccinia virus) and NS1 (influenza A virus)

**DOI:** 10.1038/s41598-019-48559-6

**Published:** 2019-08-19

**Authors:** Yi Liu, Jas Min Chin, En Lin Choo, Kyle K. L. Phua

**Affiliations:** 1 Department of Chemical and Biomolecular Engineering, 4 Engineering Drive 4, Singapore, 117585 Singapore; 2 School of Life Sciences and Chemical Technology, Ngee Ann Polytechnic, 535 Clementi Road, Singapore, 599489 Singapore

**Keywords:** Nucleic-acid therapeutics, Transfection

## Abstract

In this study, we compared vaccinia virus derived monofunctional E3, K3 and B18R (also known as EKB) with influenza A virus derived multifunctional non-structural protein 1 (NS1) based on their ability to enhance mRNA translation. EKB and NS1-TX91 were all found to enhance mRNA translation and suppress interferon production, yet level of enhancement by EKB was much lower than NS1-TX91. Similarly, greater luciferase expression was mediated by co-delivery of unmodified luciferase with NS1 mRNA, compared to co-delivery of unmodified luciferase with either E3, K3 or B18R mRNA, respectively. Different combinations of E3, K3 and/or B18R mRNA were mixed with NS1-TX91 mRNA at varying ratios and co-delivered with luciferase mRNA. However, no synergism was observed as mRNA translation enhancement mediated by NS1-TX91 could not be improved by the inclusion EKB in all tested combinations. Lastly, it was found that E3 was able to rescue mRNA translation enhancement mediated by NS1 PKR knockout mutant (PR8^PKR−^), suggesting that one of NS1’s multiple immune evasion mechanisms overlapped with E3. Altogether, our data validated mRNA translation enhancement mediated by immune evasion proteins (EKB and NS1) and showed that the multifunctional nature of NS1 accounted for its superior performance.

## Introduction

mRNA therapeutics has demonstrated great potential in recent clinical trials and preclinical studies^1,2^. Encoding antigens, growth factors, transcription factors or nucleases, *in*-*vitro*-transcribed (IVT) mRNA can be applied as vaccine against cancer and infectious diseases^1–4^, or in protein replacement therapy^5^, cell reprogramming^6^ and genome editing^7^.

IVT mRNA is able to induce antiviral responses by activating endosomal and intracellular pattern recognition receptors (PRRs), including toll-like receptors (TLR)3, TLR7, TLR8, retinoic acid-inducible gene I (RIG-I), protein kinase RNA activated (PKR) and 2′–5′-oligoadenylate synthetase (OAS)^8^. Consequently, type I interferon (IFN) is produced, leading to strengthened antiviral responses and detrimental effects including RNA degradation, global protein synthesis shutdown and cytotoxicity, all of which greatly affect mRNA’s translation capacity and reduce its biosafety^9^.

An immune evasion strategy which employs viral immune evasion proteins has been proposed to enhance mRNA translation. In nature, viruses have evolved a plethora of immune evasion proteins to counteract host antiviral responses during viral infection. By co-delivery of mRNA encoding immune evasion proteins and mRNA-of-interest, immune responses triggered during transfection are effectively suppressed, and translation of mRNA-of-interest is greatly enhanced. Such applications have been reported using vaccinia virus derived E3, K3, and B18R (EKB)^10^ and influenza A virus derived NS1^11,12^.

**Figure 1:**
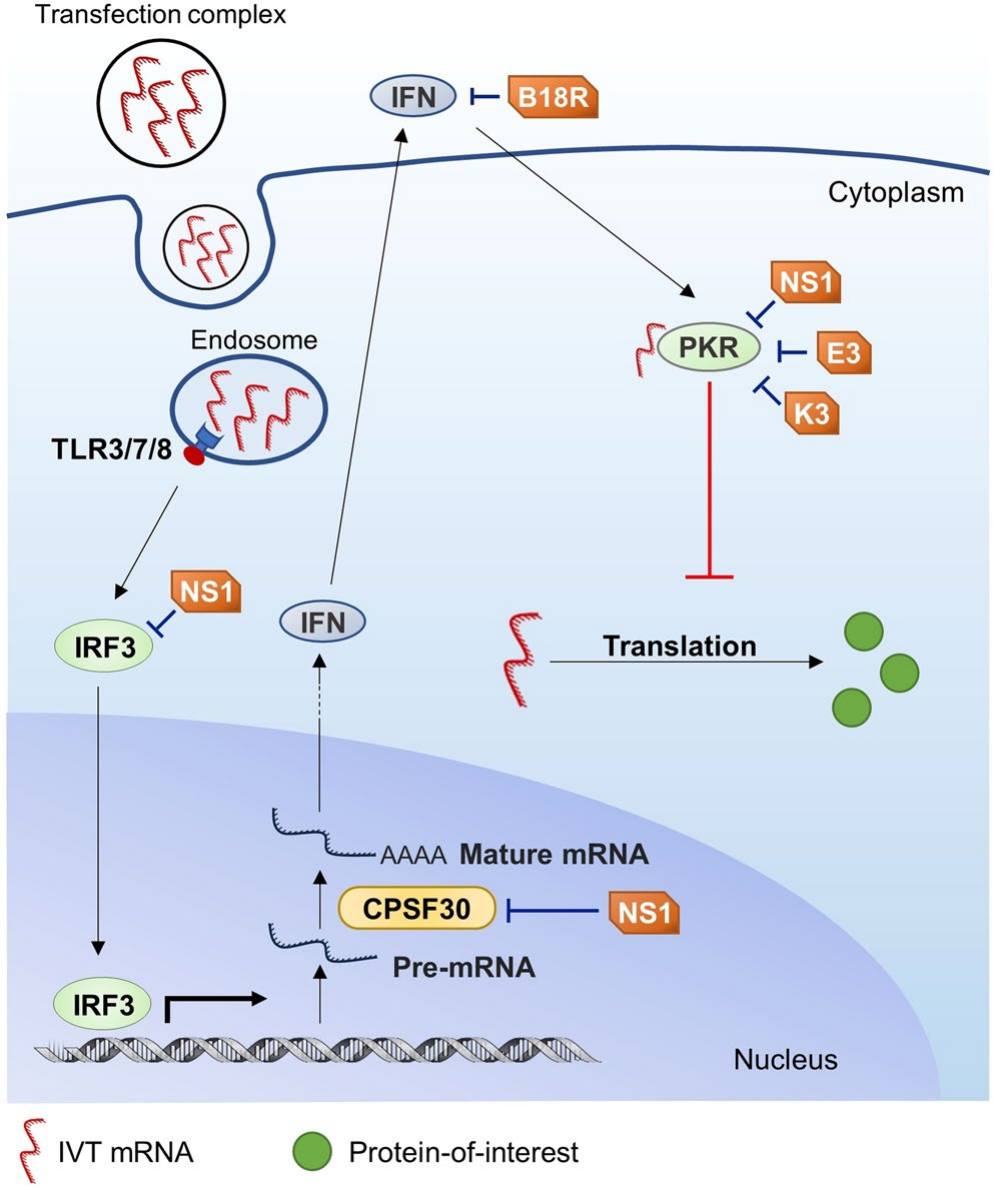
Innate immune responses triggered by IVT mRNA during transfection, and immune evasion mechanisms by E3, K3, B18R and NS1. IVT mRNA is recognized by cellular receptors such as toll-like receptors (TLR)3, TLR7, TLR8, and protein kinase RNA activated (PKR). Recognition by PKR leads to mRNA translation shutdown. Recognition by TLRs induces nuclear translocation of transcription factors such as interferon regulatory factor 3 (IRF3) and activates transcription of genes encoding cytokines such as type I interferon (IFN). Newly transcribed pre-mRNA needs to be processed to become mature mRNA and be exported out of nucleus, and cleavage and polyadenylation factor 30 (CPSF30) is indispensable for this process. Production of IFN will upregulate PKR and strengthen its effect. The vaccinia virus derived E3 and K3 inhibit PKR activation, and B18R disrupts type I IFN signalling by sequestering extracellular IFN. The influenza A virus derived NS1 inhibits PKR, IRF3, as well as CPSF30. NS1’s binding and inhibition of CPSF30 leads to accumulation of unprocessed pre-mRNA inside nucleus and disrupts global host gene expression.

E3 and K3 inhibit PKR activation, and B18R disrupts type I IFN signaling by sequestering extracellular IFN from binding to IFN-α/β receptor (IFNAR) on cell membrane (Fig. 1)^13^. NS1, in comparison, is rather multifunctional. It inhibits immune-related proteins such as PKR, OAS, interferon regulatory factor 3 (IRF3) and NF-κB, as well as non-immune related protein such as cleavage and polyadenylation factor 30 (CPSF30) (Fig. 1)^14^. NS1’s binding and inhibition of CPSF30 leads to accumulation of unprocessed pre-mRNA inside nucleus and disrupts global host gene expression, including expression of hundreds of IFN-stimulated genes (ISGs)^15^.

EKB was reported to reduce expression level of IFN-β and OAS1 in transfected cells and enhance unmodified mRNA translation^10^. NS1 was shown to enhance translation of unmodified as well as pseudouridine (ψ) and 5-methyl cytidine (m5C) modified mRNA in multiple cell types *in vitro* and *in vivo* via subcutaneous administration, and the enhancement was related to its inhibition of IRF3, PKR and CPSF30^11^.

In this study, a comparison was conducted between EKB and NS1 (subtype TX91, derived from strain A/Texas/36/91) based on their ability to enhance mRNA translation. Efficacy of these four immune evasion proteins was verified, and NS1-TX91 was found significantly more effective than EKB. Through the lack of synergism between EKB and NS1-TX91, and the rescue of PR8^PKR−^ (subtype PR8, derived from strain A/Puerto Rico/8/34, with PKR inhibition knock out) by E3, we established the superiority of NS1 owing to its multifunctionality. Finally, by comparing E3’s effect on PR8^PKR−^ and PR8^C+P−^ (subtype PR8, with CPSF30 inhibition knock in and PKR inhibition knock out), we showed the significance of host gene expression inhibition (HGEI) function in mRNA translation enhancement by NS1. These findings provide important information on application of EKB and NS1 in mRNA transfection and are of high relevance for future development of immune evasion strategy to enhance mRNA translation for the emerging mRNA therapeutics.

## Results

### NS1-TX91 mediated higher mRNA translation enhancement than EKB

**Figure 2:**
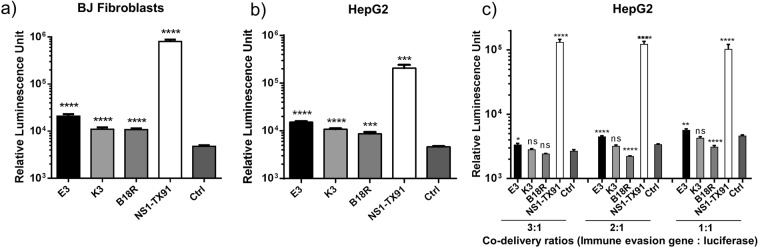
EKB could enhance mRNA translation, but not as effective as NS1-TX91. (**a**) BJ fibroblasts and (**b**) HepG2 were pretreated with pseudouridine modified E3, K3, B18R, NS1-TX91 or GFP (Ctrl) mRNA 6 h before transfection with unmodified luciferase (Luc) mRNA. (**c**) Unmodified E3, K3, B18R, NS1-TX91 or GFP (Ctrl) mRNA were co-transfected with unmodified Luc mRNA in indicated ratios (immune evasion mRNA: Luc mRNA). Luciferase assay was performed 18 hours after luciferase mRNA transfection for all experiments. Results from one representative experiment were shown here as mean ± SEM. ****p < 0.0001, ***p < 0.001, **p < 0.01.

In order to confirm that mRNA translation could be enhanced by EKB, as well as to compare their enhancement with that led by NS1-TX91, human foreskin fibroblasts (BJ fibroblasts) and human hepatocellular carcinoma cells (HepG2) were pre-transfected with pseudouridine (ψ) modified mRNA encoding E3, K3, B18R, NS1-TX91 or green florescence protein (GFP) as a control, followed by transfection with unmodified luciferase mRNA 6 h later. Human foreskin fibroblasts (BJ fibroblasts) were selected for their high relevance in cellular reprogramming which is common mRNA application. Human hepatocellular carcinoma cells (HepG2) were chosen because liver is an attractive target organ for non-viral gene therapy. The use of ψ-modified mRNA during the pre-treatment step was to avoid pre-mature activation of the cell’s immune responses against mRNA which would, in our experience, render the cells recalcitrant to a second round of transfection performed a few hours later. Expression of immune evasion protein from transfected mRNA in both ψ-modified and unmodified format was confirmed (Supplementary Fig. 1). Consistent with luciferase (Luc) expression from transfected Luc mRNA, quantified by Luc assay, expression of NS1-PR8 protein from SM mRNA was also higher than that from UM mRNA. All mRNAs used in this study are transcribed based on the same pGEM4Z-A64 template (see Materials and Methods), which would have similar protein expression levels. As depicted in Fig. 2a,b, mRNA encoding various immune evasion proteins mediated higher luciferase mRNA translation compared to GFP control. However, luciferase production mediated by NS1-TX91 was over 10 times higher than that by EKB. Among EKB, E3 mediated the highest translation enhancement, followed by K3 and B18R. No cytotoxicity was observed for all treatment groups (Supplementary Fig. 2). To evaluate the capability of EKB and NS1-TX91 to enhance mRNA translation by co-delivery, mRNA encoding each of these immune evasion proteins was transfected together with luciferase mRNA at three different ratios to HepG2 cells. Figure 2c showed consistent trend with Fig. 2a,b that NS1-TX91 mediated over 10-fold higher luciferase production than EKB for all tested ratios. Notably, B18R induced slight inhibition when its dose was increased.

### EKB and NS1-TX91 could suppress interferon production induced by mRNA transfection

**Figure 3:**
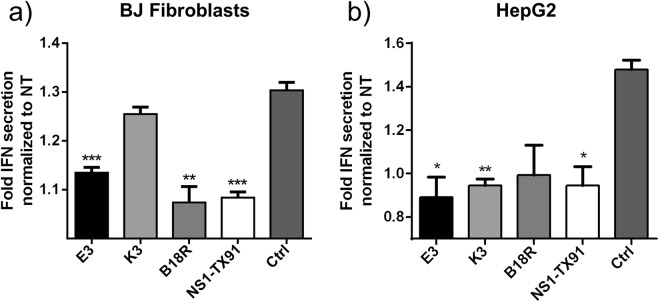
Suppression of interferon (IFN) secretion by EKB and NS1-TX91. IFN-β secretion levels 18 hours after transfection with EKB, TX91 or GFP (Ctrl) mRNA (unmodified format) in (**a**) BJ fibroblasts and (**b**) HepG2. Cells were transfected with indicated mRNA and 18 h later, supernatant was collected for ELISA. IFN-β concentration of each treatment group was normalized to NT (non-transfected) group. Results from one representative experiment of two independent repeats were shown here as mean ± SEM. ***p < 0.001, **p < 0.01, *p < 0.05).

To characterize these immune evasion protein’s influence on antiviral immune responses, supernatant of transfected HepG2 and BJ fibroblasts was collected and IFN-β was measured by ELISA. E3 and NS1-TX91 suppressed IFN secretion in both cell types, while K3 and B18R showed cell type-dependent effect (Fig. 3). Overall, it again confirmed the efficacy of EKB and TX91 in suppressing immune responses triggered during transfection.

### No synergism was observed for EKB and NS1-TX91

**Figure 4:**
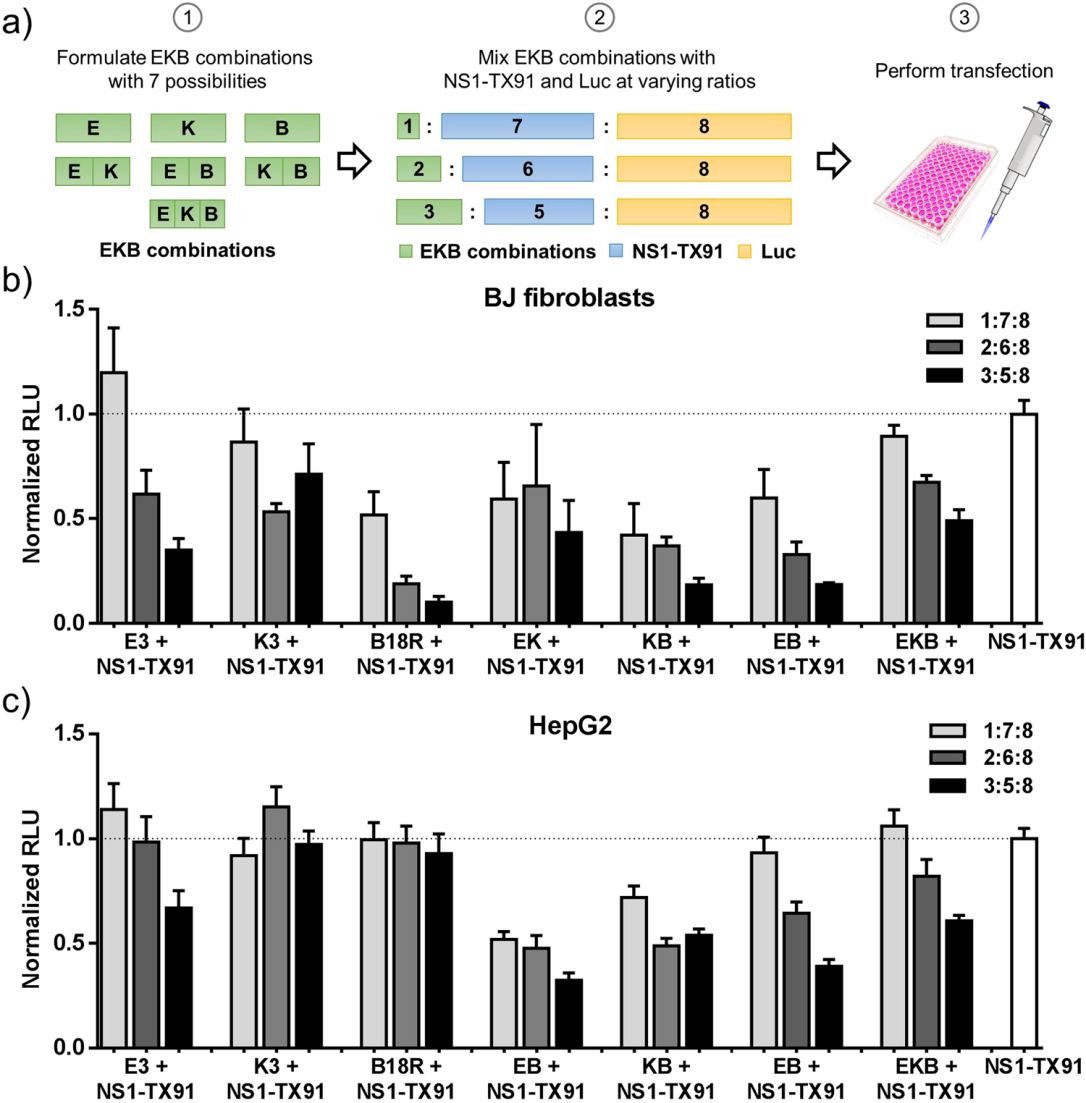
mRNA translation enhancement mediated by NS1-TX91 was not further boosted by EKB. (**a**) Dosing scheme illustration of the experiment. mRNA encoding E3 (E), K3 (K) or B18R (B) were first formed into EKB combinations with 7 possibilities, then mixed with mRNA encoding NS1-TX91 and luciferase (Luc) at three different ratios (EKB combinations: NS1-TX91: Luc = 1:7:8, 2:6:8 or 3:5:8) and transfected. Control group was transfected with NS1-TX91 contains only mRNA encoding NS1-TX91 and Luc (1:1). (**b**) BJ fibroblasts and (**c**) HepG2 were transfected with indicated combinations and ratios of mRNA. Luciferase assay was performed 18 hours after transfection. Relative luminescence units of each group were normalized to NS1-TX91 group. Results were shown here as mean of three independent experiments ± SEM.

Since immune evasion mechanisms possessed by NS1-TX91, E3, K3 and B18R are not identical, we wondered whether any synergism exists between EKB and NS1-TX91. In other words, whether individual immune evasion mechanism engendered by EKB, respectively, can synergize with NS1-TX91’s and enable higher mRNA translation enhancement than NS1-TX91 does alone. To test this hypothesis, we systematically tested all possible combinations of the three immune evasion genes (E3, K3 and B18R) with NS1-TX91. As shown in Fig. 4a, the co-delivery ratio between immune evasion mRNAs and luciferase mRNA was kept constant at 1:1, but the components of immune evasion mRNAs were adjusted as follows: First, mRNA encoding E3, K3, and B18R were formed into “EKB combinations” with 7 possibilities: one component (E3, K3 or B18R), two components with equal mass ratio (EK, EB or KB), and three components with equal mass ratio (EKB). Next, each “EKB combination” was mixed with mRNA encoding NS1-TX91 and luciferase at 3 different ratios: 1:7:8, 2:6:8 or 3:5:8 (EKB combination: NS1-TX91: luciferase), resulting in a total of 21 experimental groups. As a comparison, NS1-TX91 without any EKB was co-delivered with luciferase mRNA at 1:1 ratio. Experiments were conducted on both HepG2 and BJ fibroblasts and luciferase expression was quantified 18 h after transfection. Each group’s luciferase assay reading was normalized to that of NS1-TX91 group and depicted in Fig. 4.

In all experimental groups except for K3 + NS1-TX91 group, luciferase production decreased as the proportion of “EKB combination” increased. At low “EKB combination” proportion of E3, E3 + NS1-TX91 group reached similar mRNA translation level with NS1-TX91 alone. Taken together, it was concluded that the addition of EKB could not further uplift the mRNA translation enhancement mediated by NS1-TX91.

### E3 could rescue PR8^PKR−^ but not PR8^WT^, PR8^IRF3−^, PR8^C+P+^ or PR8^C+P−^

One possible reason for the absence of synergism between EKB and NS1-TX91 could be that NS1’s mechanism of action against PKR overlapped with that of E3 and K3. As a result, partial substitution of NS1-TX91 with E3 and K3 did not offer additional immune evasion effects and could not lead to further mRNA translation enhancement. Another possible reason was that NS1-TX91’s HGEI function mediated sufficiently potent immune evasion effects that completely outweighed those mediated by EKB and made them redundant.

**Figure 5:**
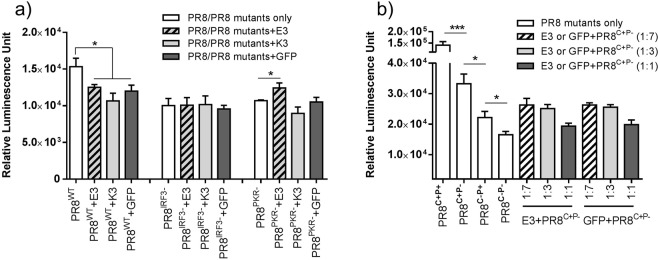
Comparison of immune evasion mechanisms of NS1, E3 and K3. (**a**) HepG2 cells were transfected with either a 1:1 combination of mRNA encoding PR8^WT^ (wild type) or PR8 mutants (PR8^IRF3−^ and PR8^PKR−^) and luciferase (Luc), or a combination of mRNA encoding E3/K3/GFP, PR8^WT^ or PR8 mutants (PR8^IRF3−^ and PR8^PKR−^) and Luc (E3/K3/GFP: PR8/PR8 mutants: Luc = 1:7:8). (**b**) HepG2 cells were transfected with either a 1:1 combination of mRNA encoding PR8 variants (PR8^C+P+^, PR8^C+P−^ PR8^C−P+^ (i.e. PR8^WT^), PR8^C−P−^ (i.e. PR8^PKR−^)) and luciferase, or a combination of mRNA encoding E3/GFP, PR8^C+P−^ and Luc at three different ratios: 1:7:8, 1:3:4, and 1:1:2 (E3/K3/GFP: PR8^C+P−^: Luc). Luciferase assay was performed 18 hours after transfection. Results from one representative experiment were shown here as mean ± SEM. ***p < 0.001, *p < 0.05. Wild type PR8 (i.e. PR8^WT^) does not have host gene expression inhibition function, thus PR8^WT^ = PR8^C−P+^ and PR8^PKR− = ^PR8^C−P−^. PR8 is the abbreviation for NS1 subtype PR8 (derived from strain A/Puerto Rico/8/34).

To test the first possibility, we employed the well-studied NS1 subtype PR8 (derived from strain A/Puerto Rico/8/34. Unlike wildtype TX91, wildtype PR8 does not engender HGEI function) and its functional knockout mutants, i.e. PR8^IRF3−^ (loss of IRF3 inhibition) or PR8^PKR−^ (loss of PKR inhibition), which we had previously reported^11^. Table 1 summarizes their amino acid substitutions and consequent changes of immune evasion mechanisms. mRNA encoding PR8 (wildtype and mutants) was either co-delivered with luciferase mRNA, or partially substituted with E3, K3 or GFP (control) and then co-delivered with luciferase mRNA. We found that addition of E3 and K3 reduced luciferase mRNA translation enhancement mediated by wild type PR8 (PR8^WT^) but had no influence on PR8^IRF3−^ (Fig. 5a). Notably, the E3 + PR8^PKR−^ combination mediated higher luciferase production than PR8^PKR−^ alone, suggesting that E3’s PKR inhibition function could complement PR8^PKR−^. However, no such observations were made with K3. Our results suggested that the lack of synergism between NS1-TX91 and E3 could be explained by overlapping mechanism of action against PKR.

**Table 1:** Immune evasion mechanisms of wild type PR8 and its mutants.

Abbreviation	Amino acid positions						IRF3	CPSF30	PKR	Ref
	38	41	103	106	123	124	R38, K41	F103, M106	I123, M124	
PR8^WT^ (PR8^C−P+^)	R	K	S	I	I	M	Yes	No	Yes	^11^
PR8^IRF3−^	A	A	S	I	I	M	No	No	Yes	^11^
PR8^PKR−^ (PR8^C−P−^)	R	K	S	I	A	A	Yes	No	No	^11^
PR8^C+P−^	R	K	F	M	A	A	Yes	Yes	No	—
PR8^C+P+^	R	K	F	M	I	M	Yes	Yes	Yes	^11^

To test the second possibility, we generated a new mutant PR8^C+P−^ (gain of host gene expression inhibition i.e. HGEI and loss of PKR inhibition, Table 1) and verified its HGEI with co-delivery with plasmid luciferase (Supplementary Fig. 3). If HGEI (being an orthogonal and non-specific mechanism) enabled sufficient immune evasion, loss of PKR inhibition would make little difference in NS1-mediated mRNA translation enhancement. As shown in Fig. 5b, when HGEI is knocked in to PR8^C−P+^ (PR8^C−P+^ and wild type PR8 are identical but with different notations, Table 1), PR8^C+P+^ mediated higher translation enhancement by an order of magnitude compared to PR8^C−P+^ as expected. However, when PKR inhibition is knocked out from PR8^C+P+^ (resulting in PR8^C+P−^, which would be similar to NS1-TX91 with loss of PKR inhibition function, Table 1), luciferase translation was higher compared to PR8^C−P+^ but significantly lower than PR8^C+P+^. The results showed that HGEI without PKR inhibition (PR8^C+P−^) could mediated higher luciferase mRNA translation enhancement than wild type PR8 (PR8^C−P+^) which has full PKR inhibition function but lacks HGEI. We further evaluated E3’s ability to restore luciferase translation by PR8^C+P−^ through partial substitution of PR8^C+P−^ with E3 at different ratios. As shown in Fig. 5b, partial substitution of PR8^C+P−^ with E3 at all ratios could not achieve statistically significant higher luciferase translation compared to respective controls (i.e. GFP + PR8^C+P−^). E3 seemed redundant when co-delivered with PR8^C+P−^ as luciferase translation was not only lower than PR8^C+P−^, but also the same as GFP control (Fig. 5b) in a dose dependent manner. Hence, even though E3 could somewhat restore luciferase translation enhancement of PR8^PKR−^ (Fig. 5a) back to PR8^WT^ level, the presence of HGEI (which is present in NS1-TX91) rendered E3’s contribution redundant.

## Discussion

mRNA as a new class of therapeutics has attracted great attention^16,17^. However, one of its limitations is its translational capacity, which is hindered by the antiviral responses triggered during transfection^8^. Recently, application of virus derived immune evasion proteins has emerged as a novel strategy to enhance mRNA translation. We and others have previously reported enhanced translation by co-delivery of mRNA encoding immune evasion proteins such as influenza A virus derived NS1^11,12^ and vaccinia virus derived E3, K3 and B18R^10^. Vaccinia virus (VV) is a DNA virus with a large genome encoding hundreds of immune evasion genes which mostly are monofunctional^13^. E3 and K3 are both VV derived PKR inhibiting proteins but act with different mechanisms. E3 protein has a C-terminal dsRNA binding region^18^ that inhibits dsRNA-dependent PKR activation by sequestering dsRNA^19^. E3 can also inhibit PKR by direct interaction^20^. K3 is a homolog of eukaryotic initiation factor 2α (eIF2α) and acts as a pseudo-substrate of PKR, thereby competitively inhibits eIF2α phosphorylation by activated PKR^21^. B18R is a secreted protein which can sequester extracellular type I IFN from binding to IFN-α/β receptor (IFNAR) on cell surface, thus disrupts paracrine and autocrine type I IFN signaling and prevents its detrimental effects^22^. In comparison with VV, influenza A virus (IAV) is an RNA virus containing only eight negative-sense, single-stranded RNA segments, which in total encode up to 17 proteins^23^. NS1, encoded by the eighth segment, performs multiple immune evasion functions including inhibition of PKR, OAS and IRF3^14^. In addition, by binding with the cleavage and polyadenylation specificity factor 30 kDa (CPSF30), NS1 disrupts nuclear pre-mRNA processing and inhibits host gene expression^14^.

B18R is the first immune evasion protein applied in mRNA transfection. In cell reprogramming studies, repeated mRNA transfection is required to reach enough production of reprogramming factors. B18R protein is applied as a medium supplement to reduce the effect of antiviral immune responses triggered by repeated transfection^24^. Polenganov *et al*. applied E3, K3 and B18R by co-delivering these mRNA together with mRNA encoding reprogramming factors, by which induced pluripotent stem cells (iPSCs) generation was achieved from human fibroblasts and endothelial progenitor cells^10^. The same group later applied them to enhance translation of self-amplifying RNA replicons *in vivo*^25^. Last but not least, we have previously reported that mRNA encoding NS1 could significantly enhance translation of both unmodified and modified mRNA^11,12^. While PKR and IRF3 inhibition contributed to translation enhancement, the key mechanism of enhancement was attributed by NS1’s ability to mediate host gene expression inhibition (HGEI) through binding with CPSF30^11^.

In this study, we compared the efficacy of E3, K3, B18R and NS1 in enhancing mRNA translation because of their different immune evasion mechanisms. NS1 from subtype A/Texas/36/91 (NS1-TX91) was selected because it engenders all three major immune evasion mechanisms contributing to mRNA translation enhancement (inhibition of IRF3 and PKR, as well as HGEI)^11^. By pretreating cells with each ψ modified mRNA encoding immune evasion proteins (E3, K3, B18R and NS1 respectively) followed by unmodified luciferase mRNA transfection (Fig. 2a,b), and secreted interferon assay by ELISA (Fig. 3), we verified their efficacy to enhance mRNA translation. Notably in our study, the level of translation enhancement achieved by EKB (Fig. 2) was not as prominent as reported in literature^10^. In comparison, NS1-TX91 showed substantially higher efficacy in enhancing mRNA translation than the rest (Fig. 2).

Given E3, K3, B18R’s unique immune evasion mechanisms, we further investigated their potential to synergize with NS1-TX91 to mediate higher translation enhancement. In literature, E3, K3, B18R were either applied alone or altogether^10,24,25^. As it was hard to predict the performance of different combinations, given the functional differences between NS1-TX91 and EKB, we included all possible combinations in a dose dependent manner so that a comprehensive evaluation was achieved. Unfortunately, none of the tested combinations could mediate higher mRNA translation than NS1-TX91 alone (Fig. 4).

NS1 has a dsRNA binding domain similar with E3^26^. It can also inhibit PKR by direct interaction^27^. The fact that E3 and K3 could not improve NS1-TX91’s performance might be due to their overlapping functions, since they share PKR as the target. Indeed, E3 was able to improve the mRNA translation enhancement led by PR8^PKR−^ but not PR8^WT^ or PR8^IRF3−^ (Fig. 5a). We also compared efficacy of NS1’s two immune evasion mechanisms: HGEI and PKR inhibition. HGEI is a consequence of NS1 protein binding to CPSF30, which prevents newly-transcribed pre-mRNA from being processed and export out of nucleus^28^. HGEI can therefore disrupt immune activation effectively because immune activation heavily relies on transcription and translation of host’s immune-related genes^14,28^. Cells’ PKR is activated by dsRNA and upregulated by type I IFN signalling pathway. Hence NS1 could not only reduce PKR activation by binding to it directly, but also reduce PKR activation indirectly by blocking immune signalling pathways that upregulate PKR expression through HGEI. Our data suggested that translation enhancement mediated by NS1’s HGEI function was more effective than its PKR inhibition function, as luciferase translation mediated by PR8^C+P−^ was higher than that by PR8^C−P+^ (i.e. wild type PR8 or PR8^WT^) (Fig. 5b). Yet, HGEI function was not potent enough to render the PKR inhibition function redundant, as luciferase translation mediated by PR8^C+P+^ was significantly higher than that by PR8^C+P−^. These observations suggested that both mechanisms synergistically enhance mRNA translation, which could not be observed between E3 and PR8^C+P−^, as co-delivery of E3 with PR8^C+P−^ (E3 + PR8^C+P−^) did not restore luciferase translation anywhere close to PR8^C+P+^ level.

In conclusion, we verified the efficacy of four viral immune evasion proteins to enhance mRNA translation: VV derived E3, K3, B18R and IAV derived NS1 (subtype TX91), and demonstrated the superior performance of NS1-TX91 in BJ fibroblasts and HepG2 cells compared with EKB. We showed that no synergism existed between EKB and NS1-TX91, and that E3 was able to improve PR8^C−P−^(i.e. PR8^PKR−^) but not PR8^C+P−^, suggesting some overlapping immune evasion mechanisms between E3 and NS1. Lastly, we showed that NS1’s host gene expression inhibition and PKR inhibition work synergistically to enhance mRNA translation. Taken together, our study provided important evidence of the promising immune evasion strategy in benefiting mRNA transfection studies and applications.

## Materials and Methods

### Cells and reagents

Human foreskin fibroblast cell line (BJ fibroblasts, ATCC) was cultured in DMEM (high glucose) growth medium supplemented with 10% heat-inactivated FBS and 1% penicillin-streptomycin. Human hepatocellular carcinoma cell line (HepG2, ATCC) was cultured in MEM growth medium (with Eagle’s basic salt solution) with the same supplements. All cells were cultured at 37 °C in a saturated humidity atmosphere with 5% CO_2_. DMEM (high glucose), MEM (with Eagle’s basic salt solution), penicillin-streptomycin 100× solution, fetal bovine serum (FBS) and trypsin 0.5% 10× solution were purchased from Hyclone, GE Healthcare Life Sciences. Alamar Blue stock solution was prepared by dissolving 1 g of resazurin sodium salt (MP Biomedicals) in 100 mL sterile PBS and filtered through a 0.22 µm filter. Stemfect mRNA transfection kit (Cat# 00–0069) was purchased from Stemgent. Steady-GLO luciferase reagent and GLO lysis buffer were purchased from Promega.

### Cloning of E3, K3 and B18R genes

E3, K3 and B18R gBlocks were synthesized by IDT. Subsequently, the gene fragments were PCR amplified and cloned into SaI-I and Not-I sites of a pGEM4Z-A64 vector containing a bacteriophage T7 polymerase promoter and 64 nucleotide-long poly A tail. All cloning was sequencing verified.

### *In vitro* transcription

*In vitro* transcription was performed as previously described^29,30^. Briefly, plasmids except for that containing B18R gene was linearized with Spe-I, purified and used as template for *in vitro* transcription using T7 High Yield RNA Synthesis Kit (Cat #E2040, NEB) in the presence of anti-reverse cap analogue (Trilink) according to manufacturer’s protocol with a capping efficiency of ~80% (based on 4:1 ratio of ARCA cap to GTP). Pseudouridine (Ψ)-modified mRNA was synthesized by the same method with uridine triphosphate completely replaced by pseudouridine triphosphate (Trilink). Plasmid containing B18R gene was linearized with Sac-I, purified and transcribed. The transcript was then subjective to enzymatic poly A tail reaction with *E*.*coli* poly (A) polymerase (Cat #M0276, NEB). Successful poly A tailling was confirmed with gel electrophoresis. All synthesized mRNA was purified with RNeasy kit (Qiagen), quantified by spectrophotometry and analyzed by agarose gel electrophoresis to confirm the synthesis of full-length mRNA.

### *In vitro* transfection

All transfection in this study was performed with Stemfect RNA transfection kit (Stemgent) according to manufacturer’s protocol. BJ fibroblasts and HepG2 were seeded at a density of 1.2 × 10^4^ cells/well on 96-well plates and incubated overnight before transfection.

For Ψ-modified mRNA pretreatment, cells were transfected with 40 ng of Ψ-modified mRNA encoding indicated genes. 6 h later, medium was replaced with fresh medium and 40 ng of unmodified luciferase (Luc) mRNA was transfected. Another 18 h later, Alamar Blue assay and Luc assay was performed.

For varying co-delivery ratio of immune evasion genes, cells were transfected with a total dose of 40 ng/well with one part being Luc mRNA and the other part being E3, K3, B18R, NS1-TX91 or GFP mRNA at indicated ratio.

For investigating synergism between EKB and NS1-TX91, cells were transfected with 40 ng/well with 20 ng being Luc and 20 ng being NS1-TX91 alone or NS1-TX91 mixed with E3, K3 and/or B18R at indicated combinations and ratios (Fig. 4a).

For exploring synergism between E3, K3 and PR8 variants, cells were transfected with 40 ng/well with 20 ng being Luc and 20 ng PR8 mutants alone or PR8 variants mixed with E3, K3 or GFP with indicated ratios.

For the above two transfection, mixed combinations and ratios were achieved by first formulating single component/gene mRNA complexes (Stemfect transfection reagent). The mRNA complexes was then diluted with culture medium and mixed based on the designed ratios and then added to cells. For all experiments, Alamar Blue assay and luciferase assay was performed 18 h after luciferase mRNA transfection. All experiments were repeated at least 3 times with the same outcome.

### Biochemical assays

Human IFN-β in the transfected cell supernatants were measured using human IFN-β ELISA kit (Elabsciences, cat # E-EL-H0085) according to manufacturer’s protocol. Cells seeded on 96-well plate were transfected with 40 ng of unmodified mRNA encoding indicated genes. 18 h after transfection, supernatant was collected (n = 3) and stored at −80 degree Celsius freezer, before ELISA was performed. Briefly, 100 μL of the supernatant (in triplicates) or human IFN-β standards (in duplicates) were added to the wells of the 96 well microtiter plate that was pre-coated with an antibody against human IFN-β. After 90 min of incubation, supernatant or standards were removed, and biotinylated detection antibody working solution was added. After another 60 min of incubation, unbound detecting antibodies were washed away with provided wash buffer and horseradish peroxidase (HRP) conjugate working solution was added. After another 30 min of incubation, solution was aspirated and subject to washing. Tetramethylbenzidine (TMB) substrate was then added and plate was incubated for 15 min in darkness. The reaction was stopped by addition of stop solution and the absorbance was measured at 450 nm with a Microplate Reader (Tecan).

To determine cell viability of transfected cells, supernatant was replaced with 100 µL Alamar Blue working solution, which was prepared by diluting Alamar Blue stock solution with fresh medium at 1:250. Alamar Blue working reagent applied at this ratio was tested on BJ fibroblasts and determined to vary linearly with cell number. After incubating for 2 h, fluorescence was measured using 544 nm excitation and 590 nm emission filter settings on a BMG LABTECH FLUOstar OPTIMA spectrophotometer.

To quantify luciferase expression, supernatant was aspirated, and cells were washed with PBS, lysed with 60 µL of Glo-lysis buffer and subjected to 3 freeze-thaw cycles. 50 μL of cell lysate was then transferred to a white opaque plate (Nunc) and mixed with 50 μL of Steady-Glo Luciferin substrate (Promega). Bioluminescence was measured by BMG LABTECH FLUOstar OPTIMA spectrophotometer.

### Statistical analysis

Results were presented as mean ± SEM. Comparisons between groups were analyzed using by multiple comparisons using one way ANOVA, followed by Dunnett’s test (experimental groups vs. control group) with GraphPad Prism. p < 0.05 was considered as statistically significant.

## Supplementary information

Supplementary Information
