# Supplementary material for: Messenger RNA translation enhancement by immune evasion proteins: a comparative study between EKB (vaccinia virus) and NS1 (influenza A virus)
Source: Sci Rep. 2019 Aug 19;9:11972. doi: 10.1038/s41598-019-48559-6 (PMC6700162; doi:10.1038/s41598-019-48559-6)
Supplement: Supplementary file 1 — Supplementary Information [file 41598_2019_48559_MOESM1_ESM.docx]

**Supplementary figures**

**Messenger RNA translation enhancement by immune evasion proteins: a comparative study between EKB (vaccinia virus) and NS1 (influenza A virus)**

Yi Liu^1^, Jas Min Chin^1^, En Lin Choo^1, 2^, Kyle K.L. Phua^1 *^

^1^ Department of Chemical and Biomolecular Engineering, 4 Engineering Drive 4, Singapore 117585, Singapore

^2^ School of Life Sciences and Chemical Technology, Ngee Ann Polytechnic, 535 Clementi Road, Singapore 599489, Singapore

*corresponding author:

Kyle K.L. Phua, Assistant Professor

Department of Chemical and Biomolecular Engineering

Faculty of Engineering, National University of Singapore

(65) 6601 3877; kyle.phua@nus.edu.sg


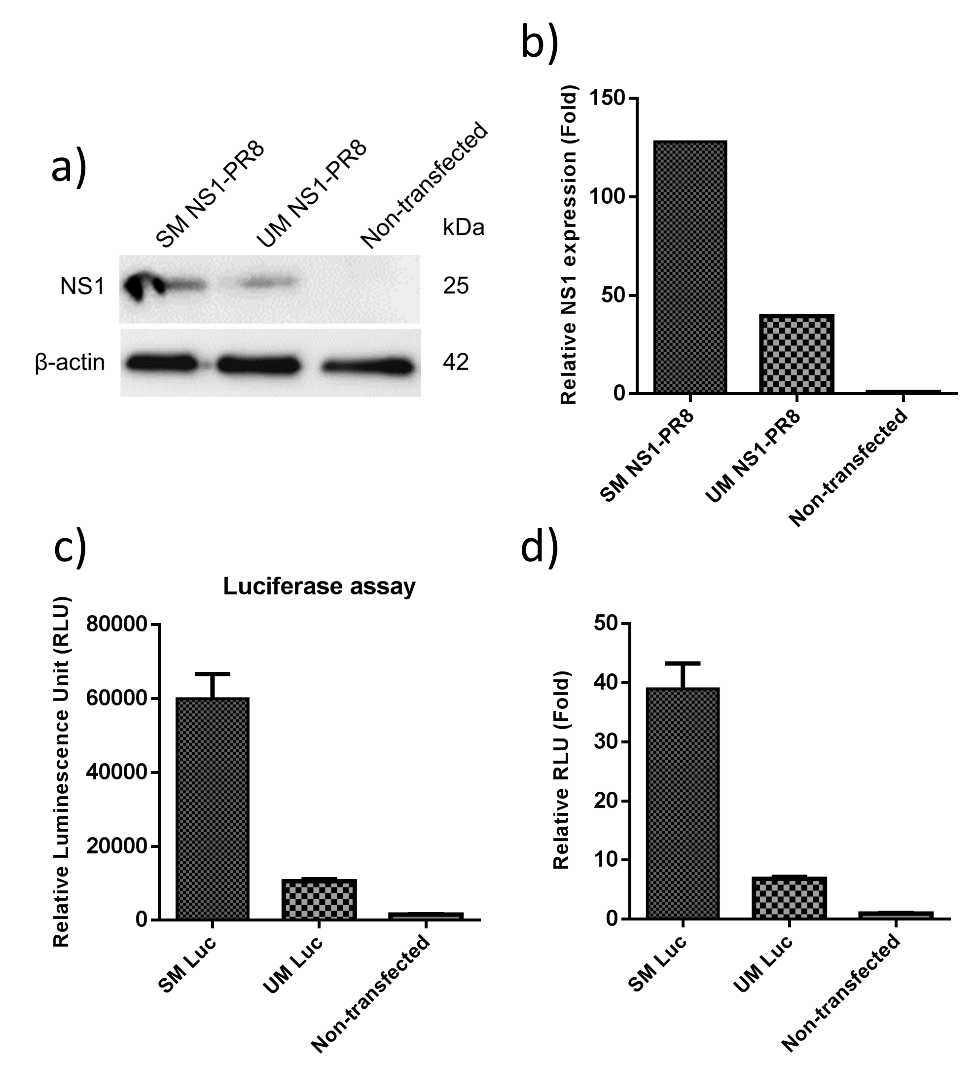


Supplementary figure 1. **Confirmation of NS1 protein expression from transfected mRNA encoding NS1.** (a) Western blot on NS1 protein. HepG2 cells was transfected with single modified (SM) or unmodified (UM) mRNA encoding NS1-PR8. 18h later, cells were harvested and western blot on NS1-PR8 and β-actin was conducted with cell lysate. (b) Semiquantitative analysis of Western blot on NS1 expression normalized to non-transfected group. (c) Luciferase assay results of HepG2 cells 18h after transfection with single modified (SM) or unmodified (UM) mRNA encoding luciferase. Results were shown here as mean $\pm$ SEM (n=5). (d) Luciferase assay results normalized to non-transfected group.


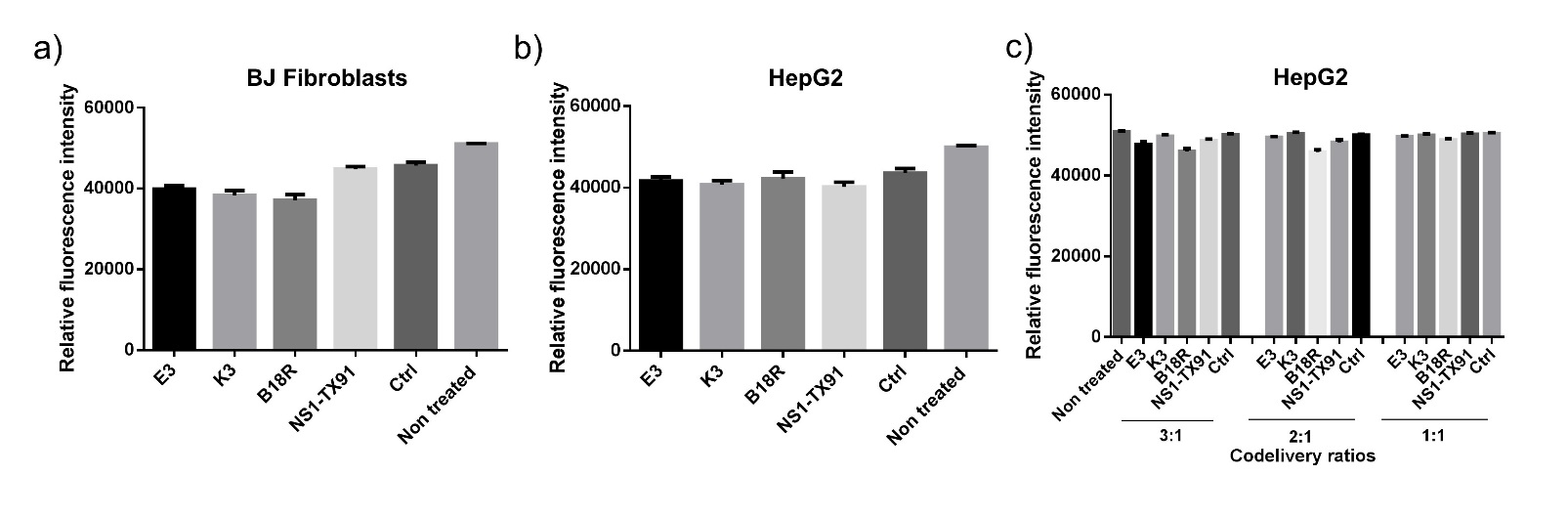
Supplementary figure 2. **Cell viability after transfection with E3, K3, B18R and NS1-TX91.** (a) BJ fibroblasts and (b) HepG2 were pretreated with pseudouridine modified E3, K3, B18R, TX91 or GFP (Ctrl) mRNA 6h before transfection with unmodified luciferase (Luc) mRNA. (c) Unmodified E3, K3, B18R, TX91 or GFP (Ctrl) mRNA were co-transfected with unmodified Luc mRNA in indicated ratios. Alamar Blue assay was performed 18 hours after luciferase mRNA transfection. Results from one representative experiment were shown here as mean $\pm$ SEM.

Supplementary figure 3. **Host gene expression inhibition of NS1-PR8 variants.** mRNA encoding PR8 variants were co-transfected with plasmid containing luciferase gene. 42h after transfection, cell viability and Luc expression was quantified. Data is presented as Luc expression normalized with Alamar Blue assay reading. Results from one representative experiment of two independent repeated were shown here as mean $\pm$ SEM.
